# GLP-1 Receptor Agonists and Dual GIP/GLP-1 Receptor Agonists in Children and Adolescents with Obesity: Clinical Outcomes and the Impact of Nutritional and Behavioral Co-Interventions—A Systematic Review

**DOI:** 10.3390/nu18111662

**Published:** 2026-05-22

**Authors:** Dominika Myśliwczyk, Krzysztof Ksawery Gofron, Andrzej Wasilewski, Małgorzata Myśliwiec, Eliza Wasilewska

**Affiliations:** 1 Department of Pediatrics, Diabetology and Endocrinology, Medical University of Gdańsk, Marii Skłodowskiej-Curie 3a, 80-210 Gdańsk, Poland; dominika.mysliwczyk@gumed.edu.pl (D.M.); malgorzata.mysliwiec@gumed.edu.pl (M.M.); 2 Student Scientific Circle at Department of Clinical Nutrition, Medical University of Gdańsk, Marii Skłodowskiej-Curie 3a, 80-210 Gdańsk, Poland; 3 Student Scientific Association of Medical Chemistry and Immunochemistry, Wroclaw Medical University, Marii Skłodowskiej-Curie 48/50, 59-369 Wroclaw, Poland; andrzej.wasilewski@student.umw.edu.pl; 4 Department of Allergology, Medical University of Gdańsk, Marii Skłodowskiej-Curie 3a, 80-210 Gdańsk, Poland

**Keywords:** pediatric obesity, adolescents, children, GLP-1 receptor agonists, semaglutide, liraglutide, exenatide, dulaglutide, tirzepatide, lifestyle intervention, nutritional counseling, systematic review

## Abstract

**Introduction:** Glucagon-like peptide-1 receptor agonists (GLP-1 RAs), originally developed for the treatment of type 2 diabetes (T2D), are increasingly used for the management of overweight and obesity in children and adolescents. However, the impact of concomitant lifestyle interventions, which vary in scope, structure, and intensity, remains unclear. **Methods:** A systematic search of PubMed, Scopus, and ClinicalTrials.gov was conducted from April to December 2025 (last update: 12 December 2025), in accordance with the PRISMA 2020 statement. Randomized and observational studies including patients aged 6–19 years with overweight or obesity, with or without T2D, treated with GLP-1 RAs or dual GIP/GLP-1 agonists, were included. Anthropometric outcomes, metabolic parameters, and the scope and structure of concomitant nutritional and behavioral interventions were assessed. **Results:** Fifteen studies (12 interventional [RCT/non-RCT] and 3 observational), including 1448 participants, were analyzed: liraglutide (*n* = 6), exenatide (*n* = 5), semaglutide (*n* = 1), dulaglutide (*n* = 1), tirzepatide (*n* = 1), and lixisenatide (*n* = 1). Intervention duration ranged from 6 to 68 weeks. Reported BMI reductions varied across studies and pharmacological agents, with semaglutide trials reporting reductions of up to −16.1%. Lifestyle interventions were heterogeneously reported, ranging from general dietary advice to structured, multidisciplinary programs including nutritional counseling, physical activity, and behavioral or family support. Due to heterogeneity in study design and reporting, the independent contribution of lifestyle interventions could not be determined. **Conclusions:** Available evidence suggests that GLP-1 RAs may represent an effective therapeutic option for children and adolescents with obesity and metabolic disorders. However, available evidence is largely derived from studies incorporating inconsistently reported lifestyle interventions, limiting the ability to disentangle pharmacological and lifestyle effects. Standardized reporting and studies specifically designed to assess their independent and combined effects are needed. Future research should standardize the reporting of lifestyle protocols (e.g., using TIDieR), incorporate validated measures of eating behavior, food preferences, and dietary intake, and use designs (e.g., factorial or stratified randomization of lifestyle intensity) that allow for the pharmacological and behavioral contributions to be quantified separately. This review highlights a critical and previously underexplored methodological gap regarding the structure and reporting of lifestyle co-interventions in pediatric GLP-1 trials.

## 1. Introduction

Pediatric obesity is one of the most pressing public health challenges of the 21st century. It is strongly shaped by dietary patterns, physical inactivity, sleep disruption, and psychosocial factors that intensify during puberty and adolescence. Over recent decades, the prevalence of excess body weight has increased markedly across both high- and middle-income countries, contributing to a growing burden of cardiometabolic disease from early life.

According to the World Health Organization (WHO), approximately 20% of children and adolescents are affected by overweight or obesity, with rates reaching 25–30% in countries such as the United States, Mexico, and Saudi Arabia [1]. In the WHO European Region, 11.4% of adolescents aged 11–15 meet the obesity criteria, while an additional 18% are overweight [2].

The etiology of pediatric obesity is multifactorial and includes poor diet quality (e.g., high consumption of ultra-processed foods and added sugars, low fiber and vegetable intake), sedentary behavior and screen time, psychosocial stress, irregular sleep, and social determinants of health. Genetic susceptibility and family history of obesity or type 2 diabetes also contribute to individual risk trajectories [3].

The health consequences of obesity during youth are broad and clinically significant. Beyond insulin resistance, type 2 diabetes, hypertension, dyslipidemia, and non-alcoholic fatty liver disease, pediatric obesity is associated with impaired quality of life, stigmatization, and mental health challenges. The increasing diagnosis of type 2 diabetes in youth is particularly concerning and highlights the accelerating impact of obesity on metabolic health at younger ages [4].

Lifestyle-based interventions remain the cornerstone of pediatric obesity management; however, achieving and sustaining clinically meaningful weight reduction in adolescents is often challenging. These limitations have driven interest in adjunct pharmacological approaches, including glucagon-like peptide-1 receptor agonists (GLP-1 RAs), which have demonstrated clinically relevant weight reduction and glycemic benefits in adults with obesity and/or type 2 diabetes [5,6].

Mechanistically, GLP-1 RAs reduce appetite and energy intake through central and peripheral pathways, including delayed gastric emptying, enhanced satiety signaling, and the modulation of reward-related eating behavior [7,8]. These effects are particularly relevant in pediatric populations, as they may relate to dietary adherence, food choice, and behavioral regulation.

Importantly, GLP-1 RAs-based therapy is rarely evaluated as a stand-alone strategy in pediatric trials; instead, it is typically embedded within broader lifestyle programs that include nutritional counseling and behavioral support. Regulatory status for incretin-based therapies in pediatric populations (approved indications and age thresholds, where applicable) varies by region and by product/dose and is summarized in Supplementary Table S1 [9,10,11,12,13,14,15,16,17,18,19,20]. Details on the specific agents evaluated in the included studies are provided in the table in Section 3.2.

Given the central role of lifestyle care in pediatric obesity and its frequent co-administration with pharmacotherapy, a key methodological challenge concerns how these co-interventions are described, structured, and reported across studies.

Importantly, the co-administration of pharmacotherapy and lifestyle interventions introduces a fundamental methodological challenge: the inability to disentangle the independent effects of GLP-1-based therapies from those of nutritional and behavioral support. This limitation has direct implications for the interpretation of clinical trial outcomes and their translation into real-world practice.

Therefore, the aim of this systematic review was to synthesize available evidence on the efficacy of GLP-1 receptor agonists (GLP-1 RAs) and dual incretin agonists in children and adolescents with overweight or obesity (including those with type 2 diabetes (T2D), and to critically examine the reporting and heterogeneity of nutritional and behavioral co-interventions across studies. To our knowledge, this is the first systematic review to specifically examine the structure, intensity, and reporting of lifestyle co-interventions in pediatric GLP-1 trials, addressing a critical and previously underexplored methodological dimension of these studies.

## 2. Materials and Methods

### 2.1. Literature Search Strategy

This systematic review was conducted in accordance with the PRISMA 2020 statement [21]. A structured literature search was carried out between April and December 2025 using the following databases: PubMed, Scopus, ClinicalTrials.gov and Embase.

The review protocol was registered in the PROSPERO database (CRD420261347002).

The aim was to identify original clinical studies, including randomized controlled trials (RCTs) and observational studies, investigating the use of GLP-1 RAs for the treatment of overweight and obesity in children and adolescents aged 6–19 years. Additionally, we manually inspected the reference lists of relevant studies and checked for any publications of interest. Data extraction was performed independently by two reviewers using predefined, standardized data extraction forms developed for the purposes of this review.

### 2.2. Search Terms and Eligibility Criteria

A systematic literature search was conducted using the following Boolean combination of keywords and Medical Subject Headings (MeSH): (“GLP-1 receptor agonists” OR “glucagon-like peptide-1 receptor agonists” OR liraglutide OR semaglutide OR exenatide OR dulaglutide OR lixisenatide OR tirzepatide)
AND(“Children and Adolescents” OR children OR adolescents OR youth OR “young people” OR pediatric OR pediatrics)AND(“Obesity” OR overweight OR “weight control” OR “weight loss”).

Only articles published in English were included. No study-design filters were applied in the search strategy to allow for the retrieval of both randomized and observational evidence. Study type eligibility was assessed during screening. The last search update was performed on 12 December 2025. The full search strategy for each database is shown in Supplementary File S1.

**Inclusion Criteria**

1. Children and adolescents aged 6–19 years with overweight or obesity, defined using age- and sex-specific pediatric criteria (e.g., WHO/CDC/IOTF cut-offs, including BMI-for-age ≥ 85th percentile for overweight and ≥95th percentile for obesity, or equivalent standardized definitions used by the original study). Studies enrolling pediatric populations with type 2 diabetes were eligible if participants had overweight/obesity or excess adiposity as a relevant clinical characteristic, as reported by the original study.
2. Intervention: Treatment with GLP-1 RAs (liraglutide, semaglutide, exenatide, dulaglutide, lixisenatide) and/or dual incretin agonists (tirzepatide), at any studied dose/regimen, alone or as add-on to standard care/lifestyle intervention.
3. Comparator: Placebo, standard care/usual care (including lifestyle counseling), active comparator, or baseline-to-follow-up comparison in single-arm interventional or observational studies.
4. Outcomes: At least one adiposity-related outcome (BMI or BMI z-score/SDS, body weight) and/or one metabolic outcome (e.g., HbA1c, fasting glucose/insulin, lipids).
5. Study design: RCTs and observational studies (prospective or retrospective cohort, registry-based studies, case–control); single-arm interventional studies were eligible if they reported extractable pre–post outcomes.
6. Follow-up: ≥4 weeks of treatment and/or follow-up.
7. Publication characteristics: full-text articles published in English.

**Exclusion Criteria**

1. Studies enrolling only adults or children < 6 years, unless data for the 6–19-year subgroup were clearly extractable.
2. Narrative reviews, systematic reviews, meta-analyses, editorials, commentaries, letters without original data, study protocols without results, and conference abstracts without sufficient extractable quantitative data, case reports.
3. Duplicate datasets or overlapping study populations (the most complete and/or most recent report was retained; earlier/partial reports were excluded).
4. Studies enrolling exclusively participants with syndromic or monogenic obesity were excluded due to distinct pathophysiology and limited generalizability to common pediatric obesity.

### 2.3. Study Selection and Data Extraction

After duplicate removal, titles and abstracts were screened independently by two reviewers (D.L., K.G.). Full-text articles were then assessed independently for eligibility by the same two reviewers. Any discrepancies were resolved through discussion, and when necessary, consultation with a third reviewer (A.W.). Extracted data included study authorship and year of publication, participant characteristics (age, sex, sample size), type and dosage of GLP-1 RAs used, study duration, primary and secondary outcomes (including changes in BMI, body weight, HbA1c, and metabolic markers), adverse events and tolerability, and descriptions of lifestyle or dietary interventions, where applicable. Data were extracted according to predefined categories. Trial registry numbers and regulatory documents (e.g., FDA approvals) were used to verify the published results where applicable.

### 2.4. Risk of Bias Assessment

Risk of bias was independently assessed by two reviewers using the RoB 2 tool for randomized controlled trials and the ROBINS-I tool for non-randomized studies. Risk-of-bias visualizations were created using the robvis tool (R package and Shiny web app) [22]. Studies were assessed across all relevant bias domains, and overall risk-of-bias judgments were assigned according to the recommended approach for each instrument. Any discrepancies were resolved through discussion, and when necessary, consultation with a third reviewer.

### 2.5. Certainty of Evidence Assessment

The overall certainty of evidence was assessed using the GRADE framework. With the inclusion of both randomized controlled trials (initially graded as high) and observational studies (initially graded as low), we assessed five domains—risk of bias, inconsistency, indirectness, imprecision, and publication bias—to determine the final strength of evidence.

### 2.6. Assessment of Nutritional and Lifestyle Components

In addition to pharmacological interventions, we also reviewed the scope and structure of nutritional and behavioral support provided in each eligible study. Particular attention was paid to whether participants received:

(1) Standardized dietary counseling,
(2) Structured physical activity recommendations,
(3) Behavioral or psychological support,
(4) Family or caregiver education.

Data were categorized according to predefined components of lifestyle intervention. This assessment aimed to determine whether GLP-1 RAs were evaluated as stand-alone interventions or in combination with lifestyle interventions. Descriptions of nutritional protocols were extracted directly from the methods sections of the published trials and their supplementary materials. Studies that lacked clear reporting on dietary components were noted as limited in this regard.

Lifestyle co-intervention intensity was classified a priori using predefined, transparent criteria based on the number and structure of reported components. This classification approach was developed specifically for the purposes of this review based on available reporting across studies. Studies were categorized as low intensity when they included only general advice without structured follow-up (≤1 component); moderate intensity when they included two structured components (e.g., dietary counseling and physical activity guidance); and high intensity when they incorporated three or more components, particularly when including behavioral/psychological support and family or caregiver involvement. Where reported, the frequency and regularity of contact (e.g., scheduled visits or counseling sessions) were considered to refine classification. Intensity categorization was performed independently by two reviewers; discrepancies were resolved through discussion, and when necessary, consultation with a third reviewer. It should be emphasized, however, that these criteria were developed pragmatically for this review and have not been formally validated against an external standard or established behavioral-intervention reporting framework (such as the Template for Intervention Description and Replication [TIDieR] or the Workgroup for Intervention Development and Evaluation Research [WIDER] recommendations). The components and thresholds therefore reflect a degree of subjective, expert-based judgment about what constitutes “structured” lifestyle support, and the resulting categorization may not be fully reproducible by independent reviewers applying different rules. For this reason, the intensity grading is presented strictly as a descriptive, hypothesis-generating heuristic rather than as a quantitative or psychometrically validated measure, and all findings derived from it must be interpreted with corresponding caution.

These differences were recorded and discussed in the synthesis of clinical outcomes, acknowledging that observed weight loss effects were not solely attributable to pharmacotherapy, but rather to a combination of interventions.

The findings were summarized in tabular form and narratively discussed to highlight patterns in clinical efficacy and implications for integration with nutritional treatment strategies. It should be noted that this classification approach is inherently limited by variability and incomplete reporting of lifestyle interventions across studies, and should therefore be interpreted as a descriptive, hypothesis-generating approach rather than as a quantitative measure.

### 2.7. Data Synthesis

Quantitative synthesis (meta-analysis) was planned if outcomes were sufficiently comparable across studies. However, due to clinical and methodological heterogeneity in populations, intervention protocols, outcome metrics, and reporting of lifestyle co-interventions, meta-analysis was not feasible. Therefore, the findings were summarized using narrative synthesis and presented in tabular form. Although several trials reported nominally similar endpoints (e.g., BMI, body weight, HbA1c), the underlying metrics and effect-size formats were not directly poolable. Specifically, BMI-related outcomes were expressed inconsistently as absolute BMI (kg/m^2^), BMI standard deviation score (BMI-SDS/z-score), or percentage change from baseline, with different reference populations used for z-score calculation. Effect estimates were variably reported as between-group treatment differences (placebo-controlled RCTs), within-group pre–post changes (single-arm and observational studies), or proportions of responders (≥5% or ≥10% BMI reduction), and the dispersion measures provided (SD, SE, 95% CI, IQR) were not uniform. Populations differed substantially in baseline indication (obesity vs. T2D with obesity), age range (6–19 years, with one trial restricted to 6–<12 years), and pubertal status. Intervention duration ranged from 6 to 68 weeks, and follow-up time-points were not aligned. The pharmacological exposures themselves were heterogeneous, comprising six different molecules (liraglutide, exenatide, semaglutide, dulaglutide, lixisenatide, tirzepatide) at different doses, frequencies, and dose-escalation schemes and lifestyle co-interventions varied widely in scope, structure, and reporting completeness, precluding any meaningful adjustment for stratification by behavioral context. Pooling under such conditions would have produced statistically combinable but clinically uninterpretable estimates and would have masked rather than clarified between study variability. The decision to abandon quantitative pooling and to rely on a structured narrative and tabular synthesis was therefore made a priori once the extent of these incompatibilities became evident at full text screening, in line with PRISMA 2020. For the same reasons, formal sensitivity and subgroup analyses could not be performed quantitatively. Instead, these elements were addressed descriptively across the narrative synthesis and risk-of-bias assessment.

## 3. Results

### 3.1. Reviewed Studies

**Figure 1:**
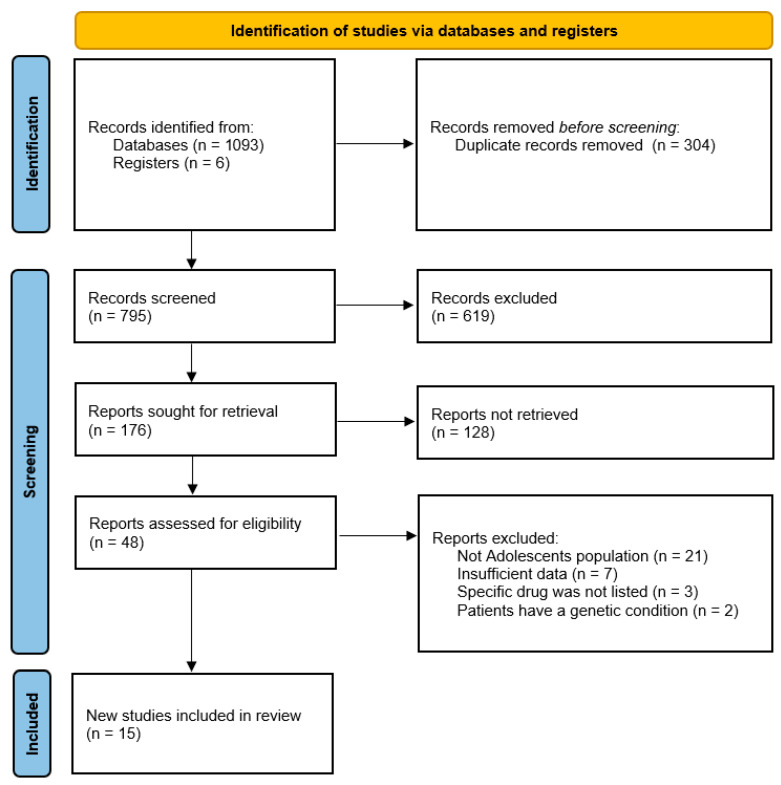
The flowchart of the search strategy.

A total of 15 studies met the inclusion criteria, comprising 12 interventional studies (placebo-controlled and non–placebo-controlled trials) [23,24,25,26,27,28,29,30,31,32,33,34] and 3 observational studies [35,36,37]. The study selection process is summarized in Figure 1.

Most studies were published from 2020 onward and differed in study design, population characteristics, intervention protocols, and duration. Although some trials included adolescents with type 2 diabetes (T2D), all participants met the criteria for overweight or obesity. Overall, substantial heterogeneity was observed across studies in terms of design, population characteristics, intervention protocols, and outcome definitions.

### 3.2. Study Characteristics

The included studies involved 1448 participants aged 6 to 19 years. Evaluated agents included liraglutide (6 studies) [32,33,34,35,36,37], exenatide (5 studies) [27,28,29,30,31], semaglutide (1 study) [26], dulaglutide (1 study) [24], lixisenatide (1 study) [25], and tirzepatide (1 study) [23]. Across the controlled interventional studies, 972 participants received active treatment and 476 were assigned to placebo groups.

Study durations ranged from 6 to 68 weeks. Included populations comprised children and adolescents with obesity [26,27,28,30,31,32,34,37] and T2D with coexisting obesity [23,24,25,29,33,35]. Details of the included studies, including population characteristics and primary therapeutic context (weight management vs. glycemic control), are presented in Table 1.

### 3.3. Risk of Bias

Risk of bias was assessed using established methodological tools selected a priori according to study design. Randomized controlled trials (RCTs) were evaluated using the revised Cochrane Risk of Bias tool (RoB 2), whereas non-randomized studies were assessed using the ROBINS-I instrument.

Each study was independently examined across all relevant bias domains, after which an overall judgment of risk of bias was assigned. Any discrepancies were resolved through discussion.

#### 3.3.1. Randomized Controlled Trials

Overall, the RCTs demonstrated variable methodological quality. Five trials [23,24,26,27,28] were classified as having a low overall risk of bias, reflecting adequate randomization procedures, good adherence to the intended interventions, complete outcome data, and appropriate outcome assessment methods.

Six trials [29,30,31,32,33,34] were judged to raise some concerns overall. The sources of these concerns varied across studies but most frequently involved missing outcome data [29,30,32,33], concerns related to the randomization process [30], deviations from intended interventions [34], or bias in the selection of the reported result [28,34].

One trial [25] was classified as having a high overall risk of bias, primarily driven by a high-risk judgment in the domain of missing outcome data, compounded by some concerns regarding both the randomization process and deviations from the intended interventions.

Across the randomized trials, bias due to missing outcome data (D3) emerged as the most frequent source of potential bias, with five studies rated as raising some concerns and one rated as high risk in this domain. Bias in measurement of the outcome (D4) was consistently rated as low risk across all included trials. Domains related to the randomization process (D1) and deviations from the intended interventions (D2) were generally rated favorably, with only two studies each raising some concerns in these domains. Although one trial was ultimately classified as high risk and several others raised some concerns, all studies were retained in the synthesis to preserve the completeness of the evidence base. However, because quantitative pooling was not possible, formal sensitivity analyses excluding studies at high or serious risk of bias could not be performed. The robustness of the observed effects to bias-related exclusions therefore remains formally untested and the strength of inferences about effectiveness must be tempered accordingly. Findings should be regarded as consistent in direction across studies of varying methodological quality rather than as quantitatively confirmed after bias restricted reanalysis.

#### 3.3.2. Observational Studies

In contrast to the randomized evidence, the observational studies exhibited a less favorable risk-of-bias profile. All three included observational studies were judged to have a serious risk of bias due to confounding (D1), reflecting the inherent methodological challenges associated with non-randomized study designs and the limited ability to adequately control for confounding factors. One study [35] received a serious overall risk of bias judgment, driven by serious risk in both the confounding and missing data domains (D1 and D5). The remaining two studies [36,37] were judged to have a moderate overall risk of bias.

Bias due to the selection of participants (D2) and deviations from intended interventions (D4) were consistently rated as moderate across all three observational studies. In contrast, the classification of interventions (D3) and measurement of outcomes (D6) were rated as low risk in all studies, indicating that intervention definition and outcome ascertainment were generally reliable. The domain of selection of the reported result (D7) yielded mixed findings, with one study rated as low risk and two as moderate risk. Nevertheless, the presence of serious bias due to confounding across all observational studies necessitates a cautious interpretation of the findings derived from this evidence.

**Figure 2:**
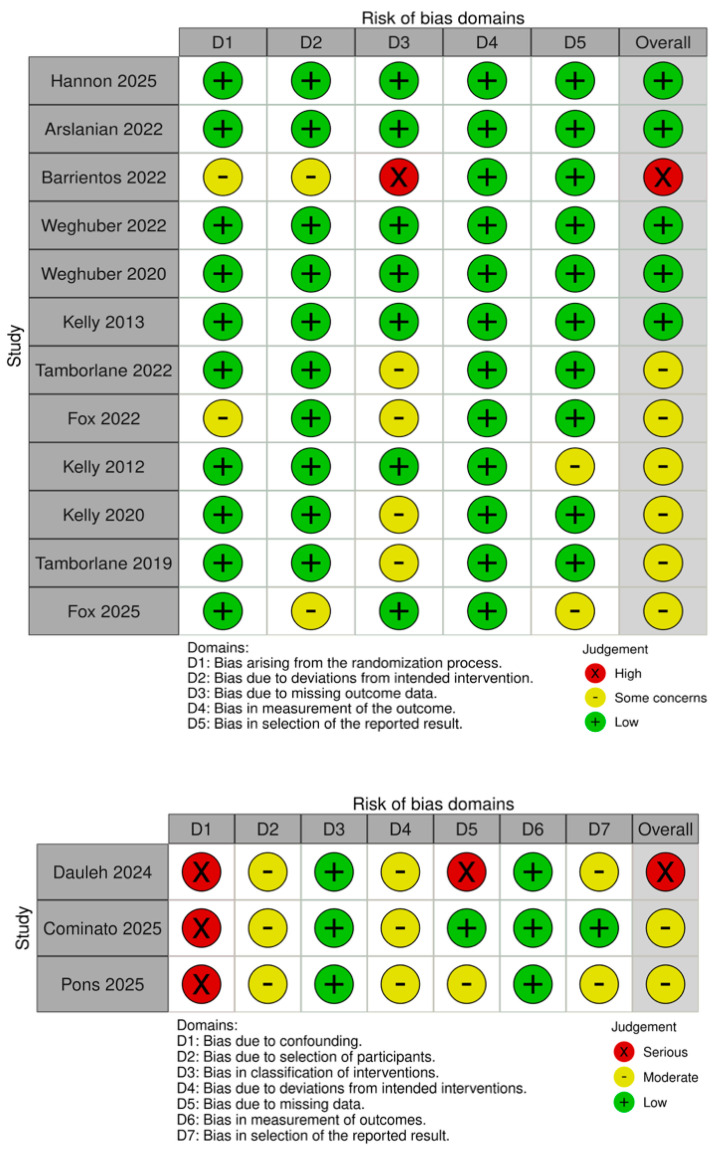
Risk of bias [23,24,25,26,27,28,29,30,31,32,33,34,35,36,37].

Overall, the predominance of moderate risk of bias among randomized trials and the presence of serious confounding in observational studies limit the strength of causal inferences and should be considered when interpreting the reported effects. Detailed domain-level assessments are presented in Figure 2.

### 3.4. Integration of Nutritional and Behavioral Interventions

Efficacy trials evaluated GLP-1 RAs in conjunction with lifestyle or behavioral components. However, the intensity, structure, and content of these interventions varied substantially, as summarized in Table 2.

Two high-quality studies, SCALE Teens [32] and STEP Teens [26], employed high-intensity multidisciplinary programs including individualized dietary counseling, caloric restriction (~500 kcal/day deficit), physical activity planning, motivational support, and consistent family involvement. Notably, these trials also reported the largest improvements in adiposity-related outcomes and metabolic parameters including high proportions of participants achieving ≥5% and ≥10% BMI reduction.

Moderate-intensity interventions typically provided structured but less frequent lifestyle support, such as standardized nutritional advice, moderate exercise guidance, and variable family participation. Examples include four studies combining basic diet and activity counseling with limited behavioral components [28,30,31,34].

In contrast, low-intensity studies offered minimal dietary advice or generalized diabetes education, with no formal behavior change support or structured follow-up [23,25,29]. These trials generally reported more variable or less consistent changes in BMI and metabolic outcomes.

Considerable heterogeneity was observed across studies with respect to both reported treatment outcomes and the structure and reporting of lifestyle co-interventions.

However, it should be emphasized that the intensity of these interventions was neither randomized nor consistently reported across studies; therefore, these observations should be interpreted as exploratory and hypothesis-generating rather than indicative of causal relationships. Moreover, the observed differences may also reflect the influence of confounding factors including variations in study design, population characteristics, follow-up duration, and pharmacotherapy regimens (see Table 2 and Table 3).

### 3.5. Body Mass and BMI Reduction

Most trials reported clinically meaningful reductions in BMI or BMI z-score (SDS), with the largest effect observed in the STEP Teens trial [26], where semaglutide resulted in a 16.1% BMI reduction. Similarly, liraglutide and exenatide trials demonstrated moderate improvements: for example, BMI decreased by approximately −0.22 SDS [32] and by −1.18 SDS [28]. The proportion of participants achieving at least 5% BMI reduction ranged from 4.92% [31] to 73% [26] depending on the intensity and structure of co-interventions.

**Figure 3:**
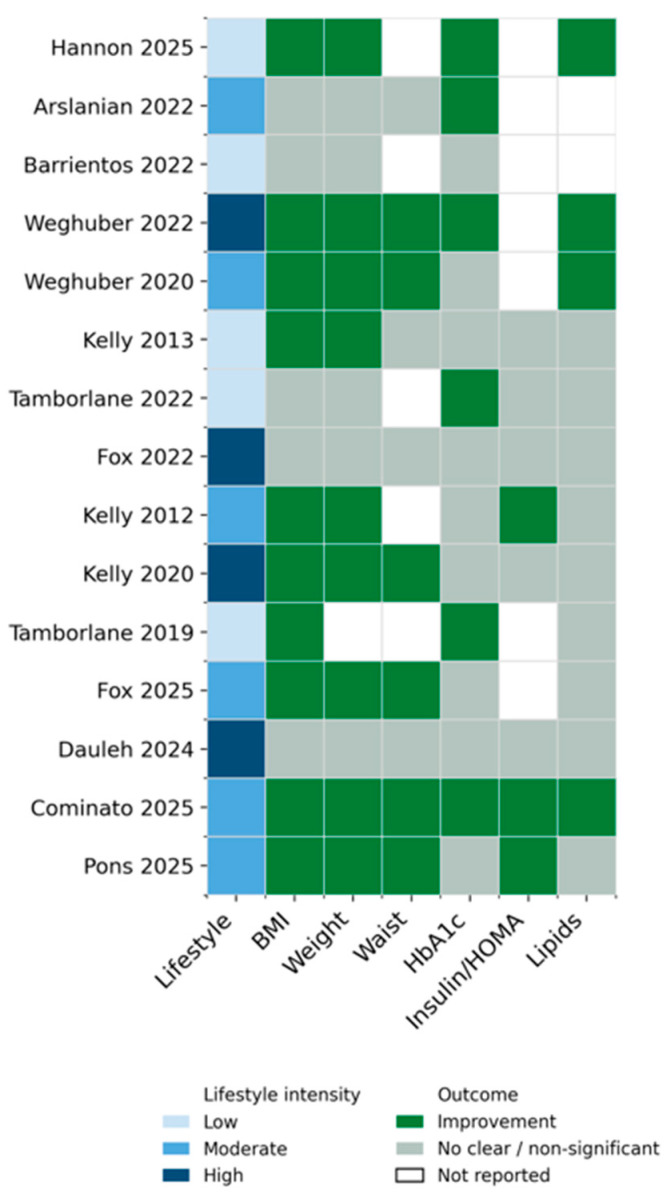
Clinical outcomes and lifestyle intensity across included GLP-1 studies. Heatmap illustrating lifestyle co-intervention intensity (blue: low intensity when they included only general advice without structured follow-up (≤1 component), moderate intensity when they included two structured components (e.g., dietary counseling and physical activity guidance), and high intensity when they incorporated three or more components, particularly when including behavior-al/psychological support and family or caregiver involvement) and the direction of clinical outcomes (green: improvement; grey: no clear effect; white: not reported). Lifestyle intensity classification was predefined and should be interpreted as descriptive [23,24,25,26,27,28,29,30,31,32,33,34,35,36,37].

A qualitative overview of lifestyle co-intervention intensity and the direction of reported adiposity and metabolic outcomes across the included studies is presented in Figure 3.

Substantial heterogeneity was observed across studies with respect to both reported treatment outcomes and the structure, intensity, and reporting of lifestyle co-interventions. Several studies reporting clinically meaningful BMI reductions also incorporated relatively detailed multidisciplinary lifestyle programs, although the independent contribution of these components could not be determined [26,30,32]. Studies incorporating less detailed or minimally reported lifestyle support also demonstrated substantial variability in weight-related outcomes across different study designs and populations [25,29]. However, because lifestyle intensity was not randomized and was variably reported across trials, these comparisons should be interpreted as hypothesis-generating rather than causal.

Among the studies that reported absolute weight change (kg), the findings also reflected this trend. In liraglutide trials, weight loss in treatment groups ranged from −2.7 to −2.3 kg, while the placebo groups varied more widely (−1.91 kg to +2.8 kg) [35]. Exenatide trials showed greater variability, with active treatment groups showing changes from −2.93 kg to −6.1 kg, and placebo groups ranging from +0.01 to +12.4 kg [28]. In the lixisenatide trial, weight increased in both groups, albeit more so in the placebo arm [25].

Reported weight-change outcomes varied substantially across studies, including those with limited or minimally described nutritional support, and should not be directly compared because of major differences in the study design, duration, and therapeutic context [25]. These observations further highlight the potential role of structured lifestyle support; however, causal relationships cannot be established based on the available data.

Unfortunately, weight change data were not reported in some key studies, including those evaluating semaglutide and dulaglutide. Nonetheless, the magnitude of the BMI and SDS reductions in these trials suggests clinically significant weight effects (see Table 3).

### 3.6. Metabolic Parameters

Beyond the anthropometric effects, several trials reported improvements in selected metabolic indices [Table 3]. Reductions in fasting insulin were reported in at least seven studies [24,26,28,31,32,33,37]. Improvements in HOMA-IR and ALT were also described in the selected trials; however, these outcomes were not consistently reported across studies and were therefore not included in the tabular summary (Table 3). Two studies measured adipokines, documenting decreased leptin and increased adiponectin, reflecting favorable changes in adipose tissue function [26,32].

However, the reporting of dietary behavior and metabolic biomarkers remained inconsistent. Recent evidence-gap work has shown that dietary intake and diet quality are rarely assessed or reported as outcomes in incretin-based trials, limiting mechanistic interpretation and the ability to link pharmacotherapy with nutrition-related behavioral change [38]. Our pediatric-focused synthesis complements this by describing how nutritional and behavioral co-interventions are implemented and reported alongside clinical outcomes in children and adolescents. Some studies reported data on lipid profiles [23,26,27,28,30,31,32,36,37], and none provided standardized measures of dietary intake or changes in food preferences. This limits the interpretation of GLP-1’s broader metabolic impact, especially in relation to nutrition-mediated mechanisms.

Table 3 summarizes the effectiveness of GLP-1 RAs in adolescents based on reported BMI outcomes, lipid changes (if available), and insulin. BMI reduction is reported either as a change in BMI (kg/m^2^), BMI z-score (SDS), or percentage change depending on available data.

### 3.7. Heterogeneity and Reporting Gaps

Considerable heterogeneity was observed in outcome measures and reporting standards across included studies. Baseline BMI was inconsistently expressed (absolute values vs. BMI-SDS), and behavioral parameters such as dietary adherence or appetite-related outcomes were rarely assessed or reported. This variability limits direct comparability between studies and obscures the nutritional context in which GLP-1-related effects occur.

The observed clinical benefits may reflect interactions between pharmacological effects and lifestyle-related factors, particularly in studies incorporating more structured interventions; however, this relationship cannot be confirmed due to heterogeneity in study design and incomplete reporting.

Furthermore, most studies did not provide sufficiently detailed data to enable the recalculation or standardization of outcomes across a uniform analytical framework. The largest gaps in reporting were identified for metabolic and hormonal parameters, including HOMA-IR, leptin, and adiponectin, which were reported in only a minority of studies.

Given their potential relevance to the underlying mechanisms of action, these parameters were retained in the review; however, they were interpreted descriptively and not used to support definitive conclusions.

## 4. Discussion

This systematic review indicates that GLP-1 RAs are associated with reductions in body mass index (BMI), improvements in selected metabolic parameters, and possibly changes in appetite-related behaviors in adolescents with obesity or type 2 diabetes (T2D). Although several trials enrolled adolescents with T2D, obesity was a near-universal characteristic across the included populations. Therefore, the findings are best interpreted in the broader context of pediatric metabolic disease rather than glycemic control alone.

Importantly, this review uniquely focuses on how lifestyle co-interventions are structured and reported in pediatric GLP-1 trials, highlighting a key methodological gap in the current evidence base.

Across the included studies, GLP-1 receptor agonists were rarely evaluated outside a broader lifestyle-care framework. Efficacy trials incorporated some form of lifestyle or behavioral support, but the intensity, structure, and content of these co-interventions varied substantially (Table 2). Two high-quality trials—SCALE Teens and STEP Teens—implemented high-intensity, multidisciplinary programs including individualized dietary counseling, caloric targets (approximately a 500 kcal/day deficit), structured physical activity planning, motivational support, and consistent family involvement [26,32]. Notably, these trials also reported the largest improvements in adiposity-related outcomes and metabolic parameters, including high proportions of participants achieving clinically meaningful BMI reductions [26,32].

Clinically meaningful BMI reductions were reported across several trials; however, substantial heterogeneity in study design, intervention structure, duration, and reporting precludes any inference regarding the independent contribution of lifestyle-support intensity to the observed outcomes. While this pattern is consistent with the hypothesis that comprehensive lifestyle support may be associated with differences in observed outcomes, lifestyle intensity was not randomized and was heterogeneously reported; between-study differences may also reflect confounding by population characteristics, intervention duration, background care, and pharmacotherapy regimen.

Studies reporting less detailed lifestyle interventions also demonstrated substantial variability in reported outcomes across different populations and study designs [29,34]. Rather than implying that pharmacotherapy is ineffective in such settings, these findings highlight that outcomes observed in pediatric trials are embedded within lifestyle care and may not be directly generalizable to settings without structured nutritional and behavioral support. This interpretation aligns with pediatric behavioral evidence showing that family-based, structured approaches—incorporating parental involvement, dietary regulation, and behavioral therapy—are among the most effective strategies for sustained weight management in youth [39,40]. It is also consistent with pediatric obesity guidelines emphasizing multicomponent care that includes diet, family engagement, and behavioral support [31,32].

Importantly, because lifestyle support was included in essentially all efficacy trials, the independent contribution of pharmacotherapy cannot be fully separated from co-interventions. This limitation has practical implications: lifestyle co-interventions should be considered when interpreting effect estimates across studies and when translating trial results into real-world care pathways.

Despite the relevance of nutritional mechanisms, most trials did not report detailed dietary intake, food preferences, or validated behavioral markers, limiting insight into how GLP-1- based therapy may interact with eating patterns and adherence. Three studies reported adipokine modulation (leptin/adiponectin) [26,31,32], and only one trial demonstrated significant lipid changes [26] constraining the interpretation of systemic metabolic impact beyond anthropometric outcomes. Given GLP-1’s central effects on appetite regulation, reward pathways, and satiety [7,41], future pediatric trials should incorporate standardized dietary assessment (e.g., dietary records/recalls with defined methodology), validated measures of eating behavior, and harmonized reporting of lifestyle protocols to enable more rigorous comparisons across interventions.

Emerging evidence outside randomized trials suggests that GLP-1 RAs may be associated with changes in eating behaviors (e.g., reduced portion size and altered preference for energy-dense foods) [42,43]. However, these observations are indirect and require confirmation in pediatric trials using standardized dietary assessment and validated behavioral measures. In addition, preclinical and early clinical research has proposed potential metabolic effects mediated through gut microbiota modulation [44], but the relevance and magnitude of this pathway in pediatric obesity remain to be established.

This review has limitations. Heterogeneity in study design, duration, populations (obesity vs. T2D), and outcome metrics restricts comparability. Lifestyle co-interventions were variably described, and detailed dietary/macronutrient data were rarely reported. Because lifestyle support was included in virtually all efficacy trials, the independent effects of GLP-1-based therapy cannot be disentangled from the co-interventions. We excluded syndromic/monogenic obesity (e.g., Prader–Willi syndrome) to improve clinical comparability and generalizability to common pediatric obesity. Definitions of overweight and obesity varied slightly across studies (percentile- vs. z-score-based criteria), which may have influenced baseline comparability. Additionally, restricting inclusion to English-language full texts may have introduced language bias. Finally, the absence of quantitative synthesis limits the ability to estimate pooled effect sizes or formally test effect modifiers, including lifestyle intensity. A further and central limitation, which warrants explicit emphasis, concerns the descriptive pattern linking higher lifestyle-intervention intensity to larger reported treatment effects. This pattern is purely observational and across-study in nature. Lifestyle intensity was not randomized within or between trials, was classified using non-validated criteria (Section 2.5), and was distributed unevenly across pharmacological agents, study designs, and populations.

The lifestyle–efficacy gradient should be read as a hypothesis to be tested in adequately designed trials with factorial or stratified randomization of lifestyle intensity, not as a causal claim. Finally, because key nutritional outcomes—dietary intake, food preferences and validated measures of eating behavior—were rarely or inconsistently reported in the primary studies, this review cannot quantify the nutritional contribution to the observed effects. To address this gap, we propose that future pediatric GLP-1 trials adopt a minimum reporting standard for the nutritional and behavioral domain, comprising: (1) a structured description of the lifestyle co-intervention using TIDieR or an equivalent framework including the planned and delivered “dose” (number, duration, and frequency of contacts; provider discipline; mode of delivery; degree of family involvement); (2) prespecified, validated measures of dietary intake (e.g., multi-day food records or 24-h recalls processed with a documented nutrient database, or a validated pediatric food-frequency questionnaire) reported as energy and macronutrient intake at baseline and follow-up. Adoption of such a minimum reporting set would substantially improve the ability of future reviews to disentangle pharmacological from behavioral contributions to weight and metabolic outcomes.

The effectiveness of GLP-1-based therapy in pediatric populations appears to be observed in the context of accompanying nutritional and behavioral interventions, the independent contribution of which cannot be determined based on the available evidence. Overall, available evidence indicates that GLP-1 RAs reduce BMI and improve selected metabolic parameters in children and adolescents with obesity, and that these effects have to date been demonstrated almost exclusively in trials that embedded pharmacotherapy within nutritional and behavioral support.

Based on our GRADE assessment, the overall certainty of evidence in the pediatric population is moderate. While the clinical effect was stable and consistent across most trials, the certainty had to be downgraded by one level due to inconsistency across studies. This inconsistency arises from the high heterogeneity in the intensity, reporting of lifestyle co-interventions, as well as structural differences that complicates the isolation of the pharmacological effect.

Future studies should better integrate and transparently report dietary and behavioral components, assess appetite-related and psychosocial outcomes, and extend follow-up to evaluate durability of benefit and long-term safety.

## 5. Conclusions

Across the included studies, GLP-1 RAs were associated with reductions in BMI and improvements in selected metabolic markers. The magnitude of observed benefits varied across trials and often coincided—descriptively and without adjustment for confounders such as drug, dose, study size, duration, or population—with the structure and intensity of co-administered lifestyle interventions, particularly structured dietary counseling and behavioral support with family involvement.

Future research should standardize the reporting of lifestyle protocols (e.g., using TIDieR), incorporate validated measures of eating behavior, food preferences, and dietary intake, and use designs (e.g., factorial or stratified randomization of lifestyle intensity) that allow for the pharmacological and behavioral contributions to be quantified separately.

## 6. Clinical Implications

In children and adolescents with obesity (including those with type 2 diabetes), GLP-1 RAs may be considered as part of a multidisciplinary approach that includes nutritional and behavioral support, in line with the design of existing clinical trials.

## Figures and Tables

**Table 1:** Summary of the study population.

Study	GLP-1 Agent	Population *n* = Active/Placebo	Age Range	Duration (Weeks)	Diagnosis	Primary Indication
Hannon et al. (2025) [23]	Tirzepatide	*n* = 130/34	10–18	30	T2D	Weight reduction
Arslanian et al. (2022) [24]	Dulaglutide	*n* = 103/51	10–18	26	T2D	Glycemic control
Barrientos-Pérez et al. (2022) [25]	Lixisenatide	*n* = 18/5	10–18	6	T2D	Glycemic control
Weghuber et al. (2022) [26]	Semaglutide	*n* = 132/64	12–18	68	obesity	Weight reduction
Weghuber et al. (2020) [27]	Exenatide	*n* = 22/22	10–18	24	obesity	Weight reduction
Kelly et al. (2013) [28]	Exenatide	*n* = 13/13	12–19	12	obesity	Weight reduction
Tamborlane et al. (2022) [29]	Exenatide	*n* = 120/0	10–18	24	T2D	Glycemic control
Fox et al. (2022) [30]	Exenatide	*n* = 33/3	12–18	52	obesity	Weight maintenance
Kelly et al. (2012) [31]	Exenatide	*n* = 12/0	9–16	24	obesity	Weight reduction
Kelly et al. (2020) [32]	Liraglutide	*n* = 125/126	12–18	56	obesity	Weight management
Tamborlane et al. (2019) [33]	Liraglutide	*n* = 66/68	10–18	24	T2D	Glycemic control
Fox et al. (2025) [34]	Liraglutide	*n* = 56/26	6–12	56	obesity	Weight reduction
Dauleh et al. (2024) [35]	Liraglutide	*n* = 37/33	12–19	52	T2D	Weight and glucose control
Cominato et al. (2025) [36]	Liraglutide	*n* = 74/0	6–18	48	obesity	Weight reduction
Pons et al. (2025) [37]	Liraglutide	*n* = 31/31	12–18	52	obesity	Weight reduction

T2D, Type 2 diabetes.

**Table 2:** Detailed description of lifestyle and nutritional interventions in GLP-1 studies.

Study	Dietary Advice	Caloric Restriction	Exercise Support	Family Involvement	Lifestyle Intensity	Lifestyle Description
Hannon et al. (2025) [23]	Yes	No	No	No	Low	Healthy meal plan
Arslanian et al. (2022) [24]	Yes	No	Yes	No	Moderate	Diet education for carbohydrate control; basic exercise support
Barrientos-Pérez et al. (2022) [25]	Yes	No	No	No	Low	General dietary advice; no formal behavioral or exercise support
Weghuber et al. (2022) [26]	Yes	Yes	Yes	Yes	High	Comprehensive program: structured diet, physical activity ≥ 60 min/day, motivational tools, parental sessions
Weghuber et al. (2020) [27]	Yes	No	Yes	No	Moderate	Reduced sugar/fat intake, dietary/exercise advice, regular check-ins
Kelly et al. (2013) [28]	Yes	No	Yes	No	Low	Short-term plan with nurse and dietitian, caloric deficit 250–500 kcal/day, exercise advice
Tamborlane et al. (2022) [29]	Yes	No	No	No	Low	General diabetes education, no structured follow-up
Fox et al. (2022) [30]	Yes	Yes	Yes	Yes	High	Weight maintenance: biweekly counseling, high-protein diet, motivational support, exercise advice
Kelly et al. (2012) [31]	Yes	No	Yes	No	Moderate	Dietary consultations and increasing levels of physical activity
Kelly et al. (2020) [32]	Yes	Yes	Yes	Yes	High	Structured multidisciplinary program: weekly counseling, 500 kcal/day deficit, daily activity, family involvement
Tamborlane et al. (2019) [33]	Yes	No	No	No	Low	General diabetes education; basic dietary and activity advice
Fox et al. (2025) [34]	Yes	No	Yes	No	Moderate	Individualized counseling to encourage adherence to healthy diet and a goal of 60 min per day of moderate to high intensity physical activity
Dauleh et al. (2024) [35]	Yes	Yes	No	Yes	High	Family-based program: dietary consultations, motivational interviewing, structured home guidance
Cominato et al. (2025) [36]	Yes	No	Yes	No	Moderate	Lifestyle counseling by a multidisciplinary team (nutritionist, psychologist, endocrinologist), activity advice
Pons et al. (2025) [37]	Yes	No	Yes	No	Moderate	Dietary advice, the total hours of physical activity per week

**Table 3:** Adiposity and metabolic outcomes following GLP-1 receptor agonist therapy in children and adolescents, reported as presented in the original studies.

Author Year	Adiposity Outcomes (as Reported in the Original Studies)	Selected Metabolic Outcomes (as Reported in the Original Studies)
Hannon 2025 [23]	BMI % −8.93 (−11.91 to −5.95), *p* < 0.0001 BMI-SDS −0.54 (−0.72 to −0.35), *p* < 0.0001	HbA1c −2.2% (−2.87 to −1.69), *p* < 0.0001
Arslanian 2022 [24]	BMI *p* = 0.5 Weight, waist circumference, NS	HbA1c −1.4% (−1.9 to −0.8), *p* < 0.001 Fasting glucose −35.9 mg/dL (−54.2 to −17.6), *p* < 0.001
Barrientos-Pérez et al., 2022 [25]	BMI %, NS Weight, NS	HbA1c, NS Fasting glucose *p* = 0.003
Weghuber 2022 [26]	BMI % −16.7 (−20.3 to −13.2), *p* < 0.001 BMI-SDS −1.1 (−1.3 to −0.8), *p* < 0.001 Weight −17.7 kg (−21.8 to −13.7), *p* < 0.001 Waist −12.1 cm (−15.6 to −8.7), *p* < 0.001	HbA1c −0.2 (−0.3 to −0.1), *p* < 0.001 Total cholesterol −7.1 mg/dL (−10.5 to −3.5), *p* < 0.001 HDL cholesterol +4.7 mg/dL (−1.0 to 10.7), *p* < 0.001
Weghuber 2020 [27]	BMI % −0.2 (−0.4 to 0.0), *p* < 0.05 BMI-SDS −0.09 (−0.18 to 0.00), *p* < 0.05 Weight −3.0 kg (−5.8 to −0.1), *p* < 0.05	Total cholesterol −11.6 mg/dL (−21.7 to −1.5), *p* < 0.05 LDL cholesterol −7.3 mg/dL (−14.2 to −0.4), *p* < 0.05 Triglycerides +8.0 mg/dL (−8.2 to 24.2), *p* < 0.05
Kelly 2013 [28]	BMI % −2.7 (−5.02 to −0.37), *p* < 0.025 BMI −1.13 kg/m^2^ (−2.03 to −0.24), *p* = 0.015 Weight −3.26 kg (−5.87 to −0.66), *p* = 0.017	HbA1c *p* = 0.072 Lipid *p* = 0.722
Tamborlane 2022 [29]	BMI % −1.22 (−3.59 to 1.15), *p* = 0.307	HbA1c −0.85, *p* < 0.012 Lipid, NS
Fox 2022 [30]	BMI % −4.8 (−10.6 to 0.9), *p* = 0.098 Weight −4.4 kg (−9.5 to 0.6), *p* = 0.087	Total cholesterol *p* = 0.462 LDL cholesterol *p* = 0.155 Triglycerides *p* = 0.213
Kelly 2012 [31]	BMI % −1.71 (−3.01 to −0.42), *p* = 0.01 Weight −3.90 kg (−7.11 to −0.69), *p* = 0.017	Insulin −7.54 mU/L (−13.71to −1.37), *p* = 0.017 Total cholesterol *p* = 0.354 LDL cholesterol *p* = 0.468 HDL cholesterol *p* = 0.266 Triglycerides *p* = 0.599
Kelly 2020 [32]	BMI kg/m^2^, NS Weight kg, NS	Total cholesterol, LDL cholesterol, triglycerides, NS
Tamborlane 2019 [33]	BMI-SDS −0.18 (−0.33 to −0.03), *p* = 0.002	HbA1c −1.3 (−1.89 to −0.7), *p* < 0.001
Fox 2025 [34]	BMI % −7.4 (−11.6 to −3.2), *p* < 0.0001 Weight −8.4 kg (−13.4 to −3.3), *p* = 0.0001	HbA1c −0.1(−0.2 to 0.0), *p* < 0.0001
Dauleh 2024 [35]	BMI *p* = 0.15 Weight kg, NS	HbA1c *p* = 0.67
Cominato 2025 [36]	Children aged 6–12 years: BMI-SDS +3.90 (3.4–5.1) to +3.06 (2.7–3.7), *p* < 0.0001 Adolescents aged >12 years: BMI-SDS +3.77 (3.02–4.66) to +3.48 (2.64–4.34), *p* < 0.0001 Weight −5.99 kg, *p* < 0.0001 BMI % −3.35, *p* < 0.0001	Children aged 6–12 years: LDL cholesterol, *p* < 0.01 HbA1c, *p* < 0.001 Adolescents aged >12 years: LDL cholesterol, *p* < 0.05 HbA1c, *p* < 0.001
Pons 2025 [37]	Weight −5.52 kg (−9.22 to −1.82), *p* < 0.05 BMI % −2.38 (−3.76 to −1.00), *p* < 0.05 BMI-SDS −0.99 (−1.58 to 0.4), *p* < 0.05	HbA1c (%) −0.13 (−0.29 to 0.03) *p* = 0.983 Basal insulin (mg/dL) −8.87 (−16.56 to −1.18), *p* < 0.05 Total cholesterol *p* = 0.527 LDL cholesterol *p* = 0.617 HDL cholesterol *p* = 0.975 Triglycerides (mg/dL) 49.15 (−81.55 to −16.74), *p* < 0.05

Data are presented as treatment differences or within-group changes, depending on the reporting in the original studies. Adiposity outcomes include body mass index (BMI), BMI standard deviation score (BMI-SDS, z-score), body weight, and waist circumference. Metabolic outcomes include glycated hemoglobin (HbA1c), fasting glucose, insulin, and lipid profile parameters. Abbreviations: BMI, body mass index; BMI-SDS, body mass index standard deviation score (z-score); HbA1c, glycated hemoglobin; LDL, low-density lipoprotein; HDL, high-density lipoprotein; NS, not significant.

## Data Availability

No new data were created or analyzed in this study. Data sharing is not applicable to this article.

## Supplementary Materials

The following supporting information can be downloaded at: https://www.mdpi.com/article/10.3390/nu18111662/s1, File S1: Search strategy for each database; Table S1: Incretin-based medicines discussed in this review: minimum age and approved indications (US vs EU/EEA).

## Abbreviations

BMI	Body mass index
GLP-1	G-like peptide-1
GLP-1RAs	Glucagon-like peptide-1 receptor agonists
PRISMA	Preferred reporting items for systematic reviews and meta-analyses
T2D	Type 2 diabetes
