# Supplementary material for: GLP-1 Receptor Agonists and Dual GIP/GLP-1 Receptor Agonists in Children and Adolescents with Obesity: Clinical Outcomes and the Impact of Nutritional and Behavioral Co-Interventions—A Systematic Review
Source: Nutrients. 2026 May 22;18(11):1662. doi: 10.3390/nu18111662 (PMC13258732; doi:10.3390/nu18111662)
Supplement: Supplementary file 1 [file nutrients-18-01662-s001.zip › File S1.pdf]

Search strategy for each database:

Pubmed; (("glucagon-like peptide 1 receptor agonists"[MeSH Terms] OR "GLP-1 receptor agonists"[tiab] OR "glucagon-like peptide-1 receptor agonists"[tiab] OR liraglutide[tiab] OR semaglutide[tiab] OR exenatide[tiab] OR dulaglutide[tiab] OR lixisenatide[tiab] OR tirzepatide[tiab]) AND ("adolescent"[MeSH Terms] OR adolescent*[tiab] OR youth[tiab] OR "young people"[tiab] OR pediatric[tiab] OR pediatrics[tiab]) AND ("obesity"[MeSH Terms] OR "overweight"[MeSH Terms] OR obesity[tiab] OR overweight[tiab] OR "weight control"[tiab] OR "weight loss"[tiab]))

Scopus: ( "GLP-1 receptor agonists" OR "glucagon-like peptide-1 receptor agonists" OR liraglutide OR semaglutide OR exenatide OR dulaglutide OR lixisenatide OR tirzepatide ) AND (adolescent OR adolescents OR youth OR "young people" OR pediatric OR pediatrics ) AND ( obesity OR overweight OR "weight control" OR "weight loss" )

Clinical trials:

Condition: Obesity OR Overweight; Intervention: GLP-1 receptor agonist OR Liraglutide OR Semaglutide OR Exenatide OR Dulaglutide OR Lixisenatide OR Tirzepatide; Age: Child (birth-17), Adult (18-64) [filtered for <19 years

Embase:

('glucagon like peptide 1 receptor agonist'/exp OR 'glp-1 receptor agonist':ti,ab OR 'liraglutide'/exp OR 'semaglutide'/exp OR 'exenatide'/exp OR 'dulaglutide'/exp OR 'lixisenatide'/exp OR 'tirzepatide'/exp) AND ('child'/exp OR 'adolescent'/exp OR 'pediatrics'/exp OR youth:ti,ab OR 'young people':ti,ab) AND ('obesity'/exp OR 'overweight'/exp OR 'weight control':ti,ab OR 'weight loss':ti,ab)
