# Supplementary material for: GLP-1 Receptor Agonists and Dual GIP/GLP-1 Receptor Agonists in Children and Adolescents with Obesity: Clinical Outcomes and the Impact of Nutritional and Behavioral Co-Interventions—A Systematic Review
Source: Nutrients. 2026 May 22;18(11):1662. doi: 10.3390/nu18111662 (PMC13258732; doi:10.3390/nu18111662)
Supplement: Supplementary file 1 [file nutrients-18-01662-s001.zip › Table S1.pdf]

**Table S1. Incretin-based medicines discussed in this review: minimum age and approved indications (US vs EU/EEA).**

| Active substance (class) | Product (indication) | United States (FDA): minimum age + indication | European Union/EEA (EMA): minimum age + indication |
|---|---|---|---|
| Liraglutide (GLP-1 RA) | **Saxenda** (obesity / chronic weight management) | **≥12 years**: chronic weight management as an adjunct to a reduced-calorie diet and increased physical activity (with pediatric-specific eligibility criteria in label). | **≥6 years** (children 6–<12 years: obesity with additional criteria; see EPAR/SmPC). |
| Liraglutide (GLP-1 RA) | **Victoza** (T2D) | **≥10 years**: adjunct to diet and exercise to improve glycemic control in T2D. | **≥10 years**: adjunct to diet and exercise in insufficiently controlled T2D; mono or add-on per SmPC. |
| Semaglutide (GLP-1 RA) | **Wegovy** (obesity / chronic weight management) | **≥12 years**: chronic weight management as an adjunct to diet and physical activity (pediatric wording in label). | **≥12 years**: weight management in adolescents with obesity and **body weight >60 kg** (adjunct to diet and activity). |
| Dulaglutide (GLP-1 RA) | **Trulicity** (T2D) | **≥10 years**: adjunct to diet and exercise to improve glycemic control in T2D. | **≥10 years**: treatment of T2D as adjunct to diet and exercise (mono/add-on per SmPC). |
| Exenatide ER (GLP-1 RA) | **Bydureon BCise** (T2D) | ≥10 years: adjunct to diet and exercise to improve glycemic control in T2D. | **≥10 years**: T2D (in combination with other glucose-lowering medicines incl. basal insulin, per SmPC). |
| Tirzepatide (dual GIP/GLP-1 agonist) | **Mounjaro** (T2D) | ≥10 years: T2D; pediatric clinical data described in label. | EU/EEA: indication extension **to ≥10 years** (EMA variation, Dec 2025); verify against latest SmPC at submission. |

T2D – type 2 diabetes, SmPC - Summary of Product Characteristics.
